# Editorial: Resistance to endocrine therapies in cancer, volume II

**DOI:** 10.3389/fendo.2024.1414392

**Published:** 2024-04-23

**Authors:** Anindita Chakrabarty, Stephen Hiscox, Prathibha Ranganathan

**Affiliations:** ^1^ Department of Life Sciences, Shiv Nadar Institution of Eminence, Delhi, NCR, India; ^2^ School of Pharmacy and Pharmaceutical Sciences, Cardiff University, Cardiff, United Kingdom; ^3^ Center For Human Genetics (CHG), Bengaluru, India

**Keywords:** breast cancer, prostate cancer, estrogen, androgen, endocrine therapy, resistance

Endocrine therapy is the standard of care for sex hormone-dependent breast, endometrial, and prostate cancer. It includes aromatase inhibitors (anastrozole, exemestane and letrozole), selective estrogen receptor modulators (tamoxifen and raloxifene), estrogen receptor antagonists (fulvestrant and toremifene), progestins (medoxyprogesterone acetate) and anti-androgens (apalutamide, nilutamide, and flutamide). Unfortunately, the development of endocrine therapy resistance is a common occurrence. Over the past two decades or so, significant progress has been made in deciphering the mechanisms of endocrine therapy resistance and identifying biomarkers to predict it. One outcome of this understanding is the inclusion of cyclin-dependent kinase 4 and 6 inhibitors (CDK4/6i) in the endocrine therapy regimen for the treatment of hormone receptor-positive advanced/metastatic and endocrine therapy-resistant breast cancer (BC). However, some patients either remain unresponsive to this combination or eventually develop resistance. The Research Topic “*Resistance to endocrine therapies in cancer, volume II”* features a collection of original research and review articles to provide readers with the most recent information on endocrine therapy resistance and potential ways to prevent/overcome it.

The first article in the collection is original research by Das et al. confirming the role of the epigenetic modulator jumonji C-domain-containing protein 6 (JMDJ6) in endocrine therapy resistance in breast cancer (BC). JMDJ6, an iron (Fe^2+^) and α-oxoglutarate (α-OG)-dependent protein with arginine demethylase and lysyl hydroxylase activities, is highly expressed in different cancers including BC. It is considered a poor prognostic indicator and a promising therapeutic target (1–4). A small molecule inhibitor of JMDJ6 has shown anti-tumor efficacy against estrogen receptor (ER)+ BC both as monotherapy and in combination with the ER antagonist fulvestrant (3). However, there is no direct evidence that JMDJ6 confers endocrine therapy resistance. The authors overexpressed JMDJ6 in MCF-7, an ER+ breast cancer cell line, compared the gene expression differences between the parental and JMDJ6-overexpressed cells and examined whether they respond differently to the ER modulator tamoxifen (Tam). While the transcriptional profile of the JMDJ6-Overexpressing cells differed significantly from the parental cells, it closely resembled that of the Tam-resistant cells. Both gain and loss-of-function studies helped the authors directly connect JMJD6 with Tam-resistance. The authors concluded by proposing JMDJ6 as a clinically relevant prognostic and predictive biomarker for endocrine therapy in BC.

The main focus of the review article by Abikar et al. is on androgen receptor (AR)-positive prostate cancer (PC). The authors mentioned the important effects of the female sex hormone estrogen on the prostate gland and the male sex hormone androgen in women. They also discussed the cell type specificity of sex hormone signaling within a given tissue. These facts, although generally overlooked, may dictate the response to endocrine therapy. Next, the authors discussed the potential effects of systemic and local balances of estrogen and androgen levels on endocrine treatment outcomes. The article concluded by pointing out that the differential distribution of AR and ER in the tumor cells and their microenvironment (TME) may also influence the success of endocrine therapy.

The review article by Yuan et al. started by mentioning the genomic, epigenomic and cellular mechanisms of endocrine resistance in ER+ BC Cells. These are ESR1 mutations or gene fusions leading to estrogen-independent transcriptional activation, DNA methylation, histone deacetylation, cell cycle alteration and rewiring of growth-promoting signaling pathways and the existence of cancer stem cells (CSCs). The authors then discussed in detail the functions of various components of the tumor microenvironment (TME). Among the cellular components of the TME, one of the most important contributors to endocrine therapy resistance is cancer-associated fibroblasts (CAFs) through their cell-autonomous and non-autonomous (secretory) functions. Next important players are different types of immune cells such as tumor-associated macrophages (TAMs), cytotoxic T cells, neutrophils, and myeloid-derived immunosuppressive cells, especially for evading anti-tumor immunity and promoting metastasis. The authors also pointed out the connection between the metabolic status of tumor cells and immune suppression and discussed in detail other cell types of TME, such as proinflammatory cancer-associated adipocytes (CAAs), vascular endothelial cells and stellate cells, which influence endocrine treatment resistance. Among the acellular components of the TME mentioned by the authors are the extracellular matrix (ECM) components produced by the fibroblasts, epithelial cells, endothelial cells and soluble factors such as cytokines, chemokines, matrix metalloproteases, and secretory vesicles (exosomes). Finally, the authors suggested the importance of considering the role of TME in improving endocrine treatment outcomes.

The article by McGrath et al. mentioned a sequential therapeutic strategy that is now adapted to avoid/overcome therapy resistance by treating BC patients with alternating anti-estrogen compounds. Despite switching the anti-estrogens in each cycle, this strategy fails even when combined with CDK4/6i. The authors speculated that two evolutionary-conserved, stress-inducible, pro-survival processes, autophagy and senescence act as adaptive mechanisms against CDK4/6i and SERMs, selective estrogen receptor downregulators (SERDs) and aromatase inhibitors. Emphasizing the importance of autophagy in endocrine therapy resistance, the authors mentioned several BC clinical trials launched to test the efficacy of autophagy inhibitors to improve response to CDK4/6i alone or with aromatase inhibitors. The focus of the review was shifted to therapy-induced senescence (to a greater extent in response to CDK4/6i than to anti-estrogens), its hallmark features such as senescence-associated secretome production and epigenetic reprogramming, mechanisms of senescence-associated induction of endocrine therapy resistance and possible ways to combat it (with senescence-targeting agents or epigenetic modulators). Although there is no concrete evidence to confirm whether senescence and autophagy interact with each other in a resistant setting, the authors pointed to a specific study in which autophagy inhibition triggered senescence in CDK4/6i-treated BC cells allowing them to adapt to a growth-arrested state. The second half of the review discussed more established modes of endocrine therapy resistance, including genetic alterations and amplifications of ESR1 and *CYP19A1* (the gene that encodes aromatase) and crosstalk between ER and growth factor signaling pathways. A lesser-known role of heat shock factor 1 in ER stability and anti-estrogen resistance was also mentioned.

In conclusion, the collection of articles discussed here emphasizes the newer aspects of endocrine therapy resistance and is likely to enhance the readers’ understanding of this topic.

## Author contributions

AC: Writing – original draft. SH: Writing – review & editing. PR: Writing – review & editing.
